# The Transition of EU Water Policy Towards the Water Framework Directive’s Integrated River Basin Management Paradigm

**DOI:** 10.1007/s00267-018-1080-z

**Published:** 2018-07-09

**Authors:** Theodoros Giakoumis, Nikolaos Voulvoulis

**Affiliations:** Centre for Environmental Policy, Imperial College London, London, SW7 2AZ UK

**Keywords:** Water policy, Integrated River Basin Management, Interdisciplinary, Holistic, Review, Implementation

## Abstract

Introduced in 2000 to reform and rationalise water policy and management across the European Union (EU) Member States (MS), the Water Framework Directive (WFD), the EU’s flagship legislation on water protection, is widely acknowledged as the embodiment and vessel for the application of the Integrated River Basin Management (IRBM) paradigm. Its ecological objectives, perhaps even more challenging than the prospect of statutory catchment planning itself, were for all EU waters to achieve ‘good status’ by 2015 (except where exemptions applied) and the prevention of any further deterioration. In support of the upcoming WFD review in 2019, the paper reviews the transition of EU policies that led to the adoption of the WFD, to identify the reasons why the Directive was introduced and what it is trying to deliver, and to place progress with its implementation into context. It further investigates reasons that might have limited the effectiveness of the Directive and contributed to the limited delivery and delays in water quality improvements. Findings reveal that different interpretations on the Directive’s objectives and exemptions left unresolved since its negotiation, ambiguity and compromises observed by its Common Implementation Strategy and lack of real support for the policy shift required have all been barriers to the harmonised transposition of the IRBM paradigm, the key to delivering good ecological status. The 2019 WFD review offers a unique opportunity to realign the implementation of the Directive to its initial aspirations and goals.

## Introduction

Water legislation is one of the European Union’s (EU) oldest, most developed and progressive areas of environmental policy (Josefsson 2012). EU freshwater policy contains several elements, but the Water Framework Directive (WFD) (Directive 2000/60/EC) is of over-arching importance. The Directive was introduced in 2000, after almost 30 years of European water legislation tackling individual issues with some considerable progress (European Environment Agency 2012), and signalled a new era of water policy. Its adoption introduced and formalised a novel approach to water management, in terms of both objectives and means (Grimeaud 2004). Establishing a common framework for water management and environmental protection based on the concept of river basin planning, the WFD has been regarded as '*the most ambitious and complex piece of legislation on environment ever enacted in the EU*' (Prieto 2009) and has been considered as a potential template and pilot for future environmental regulations (Voulvoulis et al. 2017).

**Fig. 1:**
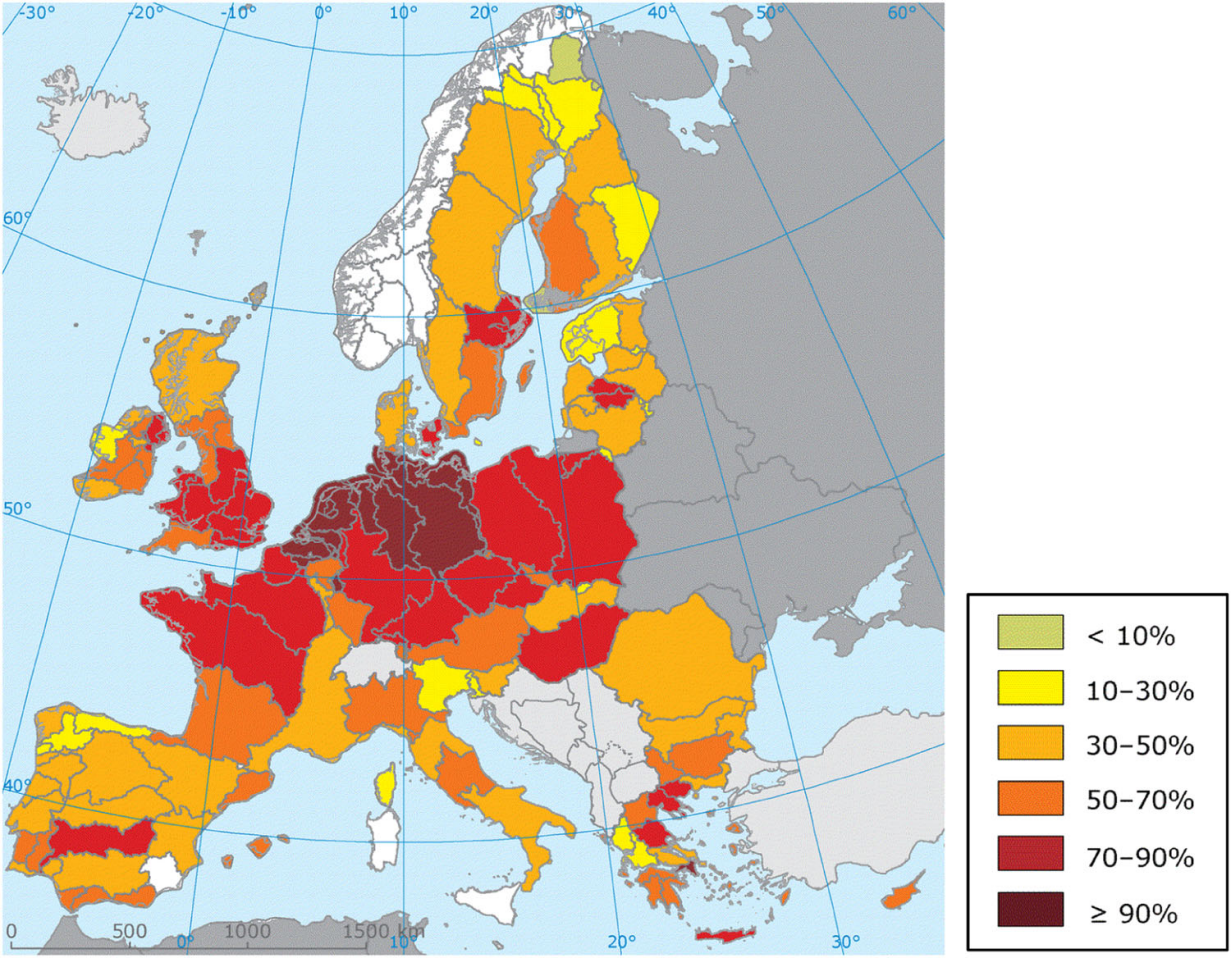
Proportion of classified river and lake water bodies in different EU River Basin Districts holding less than good ecological status or potential (European Parliament 2015)

However, despite the significant effort invested for the coordination of the WFD implementation across EU Member States (MS) and the strict timetable, the implementation process has been very challenging and progress, towards achieving the WFD objectives and improving ecological status of waters in Europe, has been slow across all MS (Fig. 1). In 2015, nearly half of EU surface waters did not reach good ecological status; the chemical status of 40% of EU water bodies was unknown (European Commission 2012a); and 73 infringement cases on non-implementation of water legislation against MS, accounting for a quarter of all environment-related infringements, were open (European Commission 2016a).

While the effectiveness of the Directive as a policy tool has been heavily criticised (Boscheck 2006; Josefsson 2012; Moss 2008) as a result of the problems above, it is in 2019 that the Commission will review the Directive (19 years after the date it was enacted) to propose any necessary amendments (Art 19). In support of this important milestone for the Directive, the paper reviews the transition of EU policies that led to the adoption of the WFD, to identify the reasons why the Directive was introduced and what is trying to deliver and to place progress with its implementation into context. It further investigates reasons that might have limited the effectiveness of the Directive and contributed to the limited delivery and delays in water quality improvements.

## The policy transition towards the WFD

**Fig. 2:**
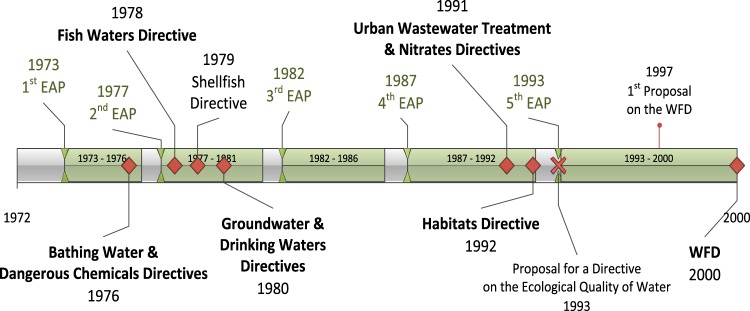
EU water policy evolution towards the WFD (1972–2000)

EU environmental policy dates back to 1972, when in the aftermath of the first UN conference on the environment, the European Council declared the need for a community environment policy flanking economic expansion and called for an action programme. This marked the start of the European Commission’s practice to periodically issue Community Environmental Action Programmes (EAP) (instruments that aim to guide the progress of Community environmental policy) that continues even today. The First EAP, covering the period 1973–1976 (Fig. 2), represents the earliest manifestation of what might be considered to be integration of the environment into other policy areas (Sheate 2003) and contained, in an embryonic form, ideas captured later by the concept of ‘sustainable development’ (Scheuer 2005). The Second EAP (1977–1981) was similar in terms of approach and objectives, advocating quality values for water, while the Third EAP (1982–1986) promoted a shift towards an emission-oriented approach.

In the period between 1975 and 1988, EU water policy focussed primarily on public health by setting Water Quality Standards (WQS) and the protection of designated water resources (Kaika 2003). It included Water Use Directives that set such standards for drinking water abstractions from surface waters culminating in the 1980 Drinking Water Directive (Council Directive 80/778/EEC), bathing waters (Council Directive 76/160/EEC), fish waters (Council Directive 78/659/EEC) and shellfish waters (Council Directive 79/923/EEC) Directives. Its main emission control element was the Council Directive 76/464/EEC on pollution caused by discharges of certain dangerous chemicals into the aquatic environment, a number of ‘daughter’ directives for specific substances and Council Directive 80/68/EEC for discharges to the ground water (Supplementary Table S1). This legislation included lists of harmful substances and set permitted levels for their discharge (specific emission limit values and quality objectives). Chemical monitoring close to point sources of pollution would check their compliance with a set of predefined standards (Petersen et al. 2009).

Although this approach was effective, it reduced environmental systems into parameters without adequate assessment of the actual environmental state and did not describe the health of aquatic ecosystems in an integrative way. Focussing on WQS for designated areas, the approach was not addressing the problems but instead was shifting them to other environmental compartments or areas (Scheuer 2005). Between 1991 and 1996, EU water legislation began focussing on the pollution emanating from urban wastewater and agricultural run-off. Legislation developed during this period was the Urban Wastewater Treatment Directive (Council Directive 91/271/EEC) and the Nitrates Directive (Council Directive 91/676/EEC), both characterised by the Emission Limit Values (ELV) approach, restricting pollutant loads allowed to be discharged into the aquatic environment. However, this approach alone was also seen as ineffective in achieving ecosystem health quality objectives (Scheuer 2005), and the Fourth EAP (1987–1992) initiated a sectoral approach, linking environmental degradation to strategic economic sectors (Scheuer 2005).

Although these EU water laws were successful for addressing specific pressures, they were looking at them in isolation (European Commission 2012b), with compliance efforts focussing on some components of the environmental system. As a result, the standard water policy was discipline-specific (England et al. 2008), with measures taken often neglecting ecosystem complexity or interdependencies across various geographical scales (Müller-Grabherr et al. 2014). Seen as incoherent (Kallis and Nijkamp 2000) as well as fragmented (Bone et al. 2011), this approach led to a recognition of the need to look at water problems more holistically.

For meeting the increasing demand on water, EU policy favoured resource development to expand supply through the public planning and funding of hydraulic infrastructures. Known as the ‘hydraulic paradigm’ or ‘hydraulic mission’ and well described in different contexts (Disco 2002; Molle 2009), it dominated bio-geographical regions affected by aridity (Del Moral et al. 2014). Through rigid management plans with little room for adaptation, uncertainty or public participation, measures were often designed and implemented on the basis of technical solutions engineered to protecting the environment rather than dealing with the source of pollution (Müller-Grabherr et al. 2014). Following the ‘command-and-control’ paradigm in management and reducing environmental systems in an attempt to make them more predictable and stable (Holling and Meffe 1996), doubts have arisen regarding the functionality of this paradigm. Policy makers started to question the potential of water quality objectives to improve the ecological quality of water bodies. In addition, setting universal quality objectives was seen as too limited as a frame of reference and policies based solely on water quality could not assure the achievement of restoration goals for freshwater systems in their entirety (Schneiders et al. 1993). Increasingly clear was the need for integration, coordination and, for systems-level, decision-making in water management problems.

A strategic reorientation was formulated with the Fifth EAP (1993–2000), which was elaborated as a response to the perceived failure of regulatory measures to achieve the Community’s environmental standards. Inspired by the Dutch National Environment Plan using a combination of regulatory, market and voluntary measures, the Fifth EAP attempted to extrapolate this approach to the Community level (FERN 1998). New regulatory approaches were promoted, which explicitly aimed to take nationally diverse conditions into consideration (Holzinger et al. 2006). In addition, with the subsidiarity principle being a general principle of action with the Single European Act (1986) and the Maastricht Treaty (1992), interventionist models were increasingly becoming politically less legitimate. The Fifth EAP set the vision for the integrated management of freshwaters.

In 1993, the proposal for a Directive on the Ecological Quality of Water (COM 93 680) was a first attempt and a big step towards this direction, as later acknowledged by the European Parliament (STOA 1995). It was proposing a '*framework for MS to improve the ecological quality of all surface waters by taking measures to control pollution from point and diffuse sources, as well as other anthropogenic factors affecting water quality so as to maintain and improve the ecological quality of Community surface waters with the ultimate aim of achieving good ecological quality*' (European Commission 1993). Some of its definitions capture better the ecological intentions of the WFD and help with the interpretation of some of the ambiguous terms used in the Directive 7 years later. For example, the proposal offers a '*procedural approach allowing the elaboration of solutions tailored to the needs in individual waters*' and acknowledging the ecological variability across different regions of the Community, the ecological quality defined by qualitative terms leaving to MS the '*specifications and the adaptation to local conditions of ecological quality for individual surface waters*'. Waters of ‘high ecological quality’ were defined as those which are not '*significantly influenced by human activities*' and of ‘good ecological quality’ when the '*self-purification of the water body is maintained, the diversity of naturally occurring species is preserved and the structure and quality of the sediments are able to sustain the naturally occurring biological community of the ecosystem*' (European Commission 1993), with a list of relevant elements determining ecological quality given (Annex I). The purpose of the proposal was to create the necessary framework to make MS define and implement measures to obtain good ecological quality. ‘Benefits’ were identified as: '*increased possibilities for recreational use by the local population, conservation of nature values and species, increased tourism potential, improving the potential for fishery and, for fresh water the qualitative and quantitative improvement of an important resource for the production of water suitable for drinking, agricultural, industrial and recreational use and other uses essential for human and economic activity*' (European Commission 1993), providing a clear reference to what we today call *ecosystem services*.

In the mid-1990s, supported by the emergence of integrated watershed management (International Conference on Water and Environment in Dublin, the United Nations Conference on Environment and Development in Rio de Janeiro, both in 1992), the thrust towards river basin management gained momentum in the EU, and the need for an overall framework to manage freshwater resources was established (Hooper 2005). Pressure to re-think European water policy soared in 1995 when the Commission accepted requests from the European Parliament’s Environment Committee and from the Council of Environment Ministers (Hooper 2005). After a widespread consultation process and a conference in May 1996, the need to overcome fragmentary water policy and establish a single piece of framework legislation emerged (Hooper 2005). The terms ‘watershed management’, ‘catchment-based management’ or ‘Integrated River Basin Management’ (IRBM), all referred to an approach in natural resources management that considers land and water as one interconnected system, in which the solutions emerge through a process of integrating environmental, economic and social aspects.

In 1996, a Communication of the European Commission on the water policy of the Community called for a Framework Directive (European Commission 1996) in order to '*concentrate, rationalise and standardise, as well as improve the efficiency of European water protection legislation*' (Dworak et al. 2007). Nearly a decade since the Council identified for a first time the need for a more comprehensive water legislation in 1988 and several interim steps, the Commission finally published its proposal for a WFD (COM (97) 49) in February 1997, replacing the Ecological Quality of Water proposal (European Commission 1997). In the WFD proposal, the main elements of the Ecological Quality of Water proposal (COM 93 680) had remained but its scope had been expanded to include groundwater resources, to deal with issues on water quantity, develop a clearer ‘framework’ and clarify its relationships with other water policies. Considered as the vessel for the implementation of the IRBM paradigm in Europe, the WFD required the coordination of management actions within River Basin Districts (COM (97) 49) following a *combined* approach of WGS and ELV.

Through the co-decision process (between the European Parliament and the European Council of Ministers) that was intensive and complicated and after 2 years of intense political negotiation and compromise (Kaika and Page 2003), the WFD was finally published, coming into force on 22 December 2000. Regarded as the most significant piece of European water legislation to be produced for over 20 years, the WFD has been widely recognised as the 'constitution' of water-related legislation in the European Union aiming to deliver a revolution away from the conventional sector-based strategies towards IRBM (Cao and Warford 2006; Solimini et al. 2009; Valinia et al. 2012).

**Fig. 3:**
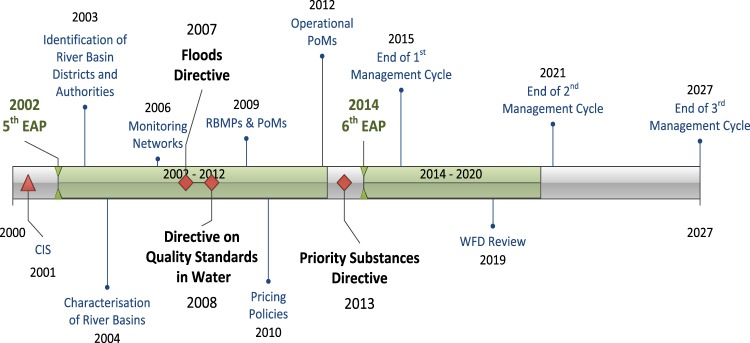
WFD implementation milestones and policy developments since its adoption (2000–2027)

The introduction of the WFD followed a period of additional policies aiming for a transition to a water-efficient and water-saving EU economy (Fig. 3). The Communication, ‘Addressing the challenge of water scarcity and droughts’ (European Commission 2007a), and the White Paper on ‘Adapting to climate change: towards a European framework for action’ (European Commission 2009a) widely recognised the water quality and availability concerns faced by many regions. In 2007, the adoption of the Floods’ Directive 2007/60/EC required alignment with the WFD’s RBM planning process as a means for concerted management action against pressures (European Parliament 2007), and in 2008, in the face of the financial, economic and social crisis, the new EU strategy promoted sustainable water management as part of the broader goal of the ‘green economy’, promoted by the EU to deliver improvements in resource efficiency, resilient ecosystems and human wellbeing. The EU Biodiversity Strategy 2020 (European Commission 2011a), the EU Resource Efficiency Roadmap (European Commission 2011b), the European Innovation Partnership on water, the ‘Blueprint to safeguard Europe’s water resources’ (European Commission 2012c) and the Seventh EAP (Decision No 1386/2013/EU) are some of the initiatives that followed (More information is given in Supplementary Table S2.).

## The WFD Paradigm Shift

**Fig. 4:**
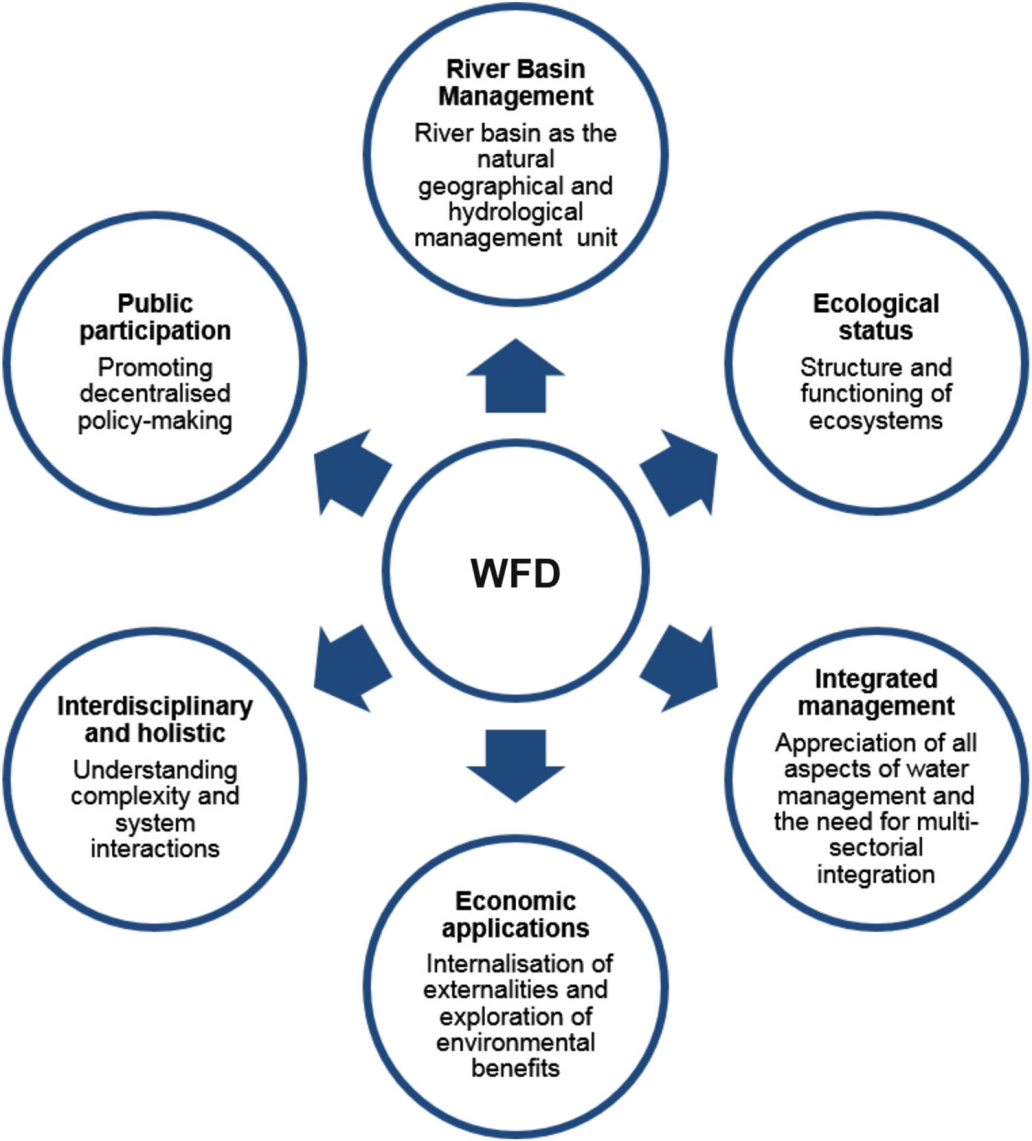
Some of the innovations introduced by the WFD

The introduction of the WFD was an evolutionary policy response to water management challenges in the EU and its adoption was received with great expectations (Chave 2007). The many innovations it introduced (Fig. 4), created a revolutionary prestige for the Directive, which required the fundamental restructuring of competencies in water management and environmental protection (Bielsa and Cazcarro 2015; Richter et al. 2013). Offering a new framework for the assessment, management, protection and improvement of the quality of water resources (Solheim et al. 2012), the WFD demanded a philosophically new approach (Bouleau and Pont 2015; Brack et al. 2009; Carter 2007; Correljé et al. 2007; Johnson 2012; Kelly 2014; Petersen et al. 2009; Pollard and Huxham 1998).

MS were required to prevent deterioration of the quality of waters and achieve good water status by managing water resources effectively through the integrated management of the wider environmental system (Bone et al. 2011; Chon et al. 2010). For this, the Directive introduced river basin management planning, an objective setting process allowing improvements to the water environment to be prioritised over successive planning cycles while ensuring that the needs of water users and other stakeholders are properly considered in decision-making (Baaner 2011; Huitema et al. 2009; van Ast and Boot 2003; Wright and Fritsch 2011). Acknowledging the ecological variability of European waters and treating the river basin, as one interconnected system, adopting its natural and hydrological boundaries, rather than political and jurisdictional ones, it introduces ‘ecological status’ as an expression of the quality of the structure and functioning of surface water ecosystems (Grizzetti et al. 2017). Its ecological objectives were for all EU waters to achieve ‘high or at least good ecological status’, defined as the state of the water ecosystem in the absence of any anthropogenic pressures or a slight biological deviation from what would be expected under undisturbed/reference conditions, respectively (Voulvoulis et al. 2017). Ecological status is determined in terms of the quality of the biological community, the hydro-morphological and physio-chemical characteristics, with a classification scheme providing an indication of the state of the aquatic environment and for assessing the effectiveness of the Programmes of Measures (PoMs) to improve its state (European Communities 2003a). This requires robust understanding of the essential components of the system and their interactions (including pressure-impact and economic analysis), to take appropriate actions to reduce pressures and improve its overall state (European Communities 2003b). Having a multidisciplinary and multi-agent approach and sharing of information (Bielsa and Cazcarro 2015), river basin management under the WFD is decentralised, participatory and inclusive of socioeconomic aspects in the integration of economic analyses of water use. The aquatic system has social and economic dimensions that must be adequately integrated in the overall decision-making process (Vugteveen et al. 2006).

The WFD was one of the first attempts of the EU to introduce governance characterised by an experimentalist architecture (Behagel and Arts 2014; Sabel and Zeitlin 2012, 2008), an iterative, open-ended framework with goals jointly established by ‘central’ (EU) and ‘local’ (MS) institutions, typically in consultation with relevant stakeholders (Zeitlin 2016). Facilitated by operational and technical obligations for its implementation by the MS, the process was supported with policy guidelines refined on the regional level via multi-country dialogue through the Common Implementation Strategy (CIS) and public participation, on top of the reporting requirements introduced by the Commission to monitor implementation progress (WFD Articles 15 and 18). The CIS was initiated in 2001 and had an informal and voluntary nature with the Strategic Co-ordination Group and the various Working Groups producing guidance documents, which were however non-binding (Scott and Holder 2005). Public involvement as a means for including all different perspectives offers a way for addressing water management complexity (Steyaert and Ollivier 2007) and plays a key role to the successful implementation of the Directive (Preamble 14).

## Implementation Problems and Delays

There is a consensus among EU water stakeholders that, despite a lot of efforts invested by the MS to implement and enforce the WFD, overall progress with implementation fell behind expectations. As early as 2007, the Commission started raising concerns (Supplementary Table S2) (European Commission 2007b). The *Third implementation report* revealed that the first RBMPs across most MS (2009–2015) were characterised by significant gaps. The gaps in monitoring of the chemical status of surface water bodies were highly significant to the extent that in 2009 for 40% of them the status was unknown and as a result no baseline was established (European Commission 2012d). Not all priority substances were monitored and the number of water bodies being monitored was very limited (Brack et al. 2017). Approximately in 15% of surface EU water bodies the ecological status was unknown (European Commission 2009b), and significant gaps remained in relation to the pressures and impact analysis (74% of MS), the development of appropriate assessment methodologies (sensitivity level to pressure) (85% of MS) and the monitoring of water status (81% of MS) (European Commission 2015). The *Fourth implementation report* revealed that the pressure assessments, the pressure–impact analysis and the source apportionments had been inefficient, weak and unreliable in 11, 14 and 15 out of the 27 MS respectively (Table 1) (European Commission 2015). In 21 out of 27 MS, clear links between pressures and measures were missing while the gap analysis had been ineffective in 23 out of the 27 MS for the development of suitable and cost-effective PoMs. Instead it seemed that most MS adopted a ‘business as usual’ approach, supported by the fact that they had only assessed how far existing measures could contribute towards WFD’s objectives. Exemptions were applied widely and often lacking adequate justifications, and in many MS, the cost-effectiveness analysis in support of the appraisal and selection of PoMs was missing (for example, post socialist EU countries) or had serious information gaps (like Greece, Spain) or certain limitations (UK, Italy) (European Commission 2015). Another key implementation problem for most water authorities was that river basin environmental objectives were not set wide enough to integrate with other policies or in some cases were incoherent or even in conflict with other policies (European Commission 2015).

**Table 1:** A summary of the implementation problems for the Member States based on the Fourth implementation report (European Commission 2015)

Implementation progress	Number of Member States (27 in total)
*Monitoring and assessment*	
• Gaps and delays in the implementation of monitoring and RBMPs	18
• Improve methodologies for status assessments	17
• Determine and finalise the reference conditions	8
• Revise, improve and make transparent the designation process of the heavily modified and artificial water bodies	10
*Pressures*	
• Improve pressure analysis	11
• Weak pressures and impacts analysis	14
• Establishing clear links between pressures and measures (improving the pressures and impact analysis for developing PoMs)	21
• Apportion pressures to relevant sources and sectors and drivers (including the need for quantitative methods)	15
*Integration of policies*	
• Need for better integration of other EU Directives and other legislative drivers in implementing the WFD	20
*Gap analysis*	
• Assess the gaps and effectiveness of basic measures	9
• Justify and set out clearly the need for supplementary measures	13
• Improved gap analysis to inform the PoMs for the achievement of objectives	23
• Providing more information regarding the scope of the measure (extent, cost of measures and expected impact on water bodies)	9
*Exemptions*	
• Improve the approaches in the application of exemptions in RBMPs	9
• Ensure that exemptions for not achieving objectives are adequately justified	20

Furthermore, in 2015, there were 73 open infringement cases on non-implementation of water legislation against MS; accounting for a 25% of all infringements in the domain of environmental policy (European Commission 2016b). The key main failures for these related to the publication of RBMPs, the lack of information and consultation of the public on the envisaged management plans and the transposition of certain articles of the WFD into the national policy context. A summary of the infringement cases that have been related to WFD implementation is provided in Supplementary Table S3.

## What went Wrong?

From the start, it was recognised that the Directive was very complex and would clearly pose many challenges (Pollard and Huxham 1998; Quevauviller et al. 2005; WWF and EEB 2004). While the importance of governance systems in delivering efficient water management and the effective implementation of the WFD is widely recognised, even acknowledged by the Commission (European Commission 2012e), the Directive did not address the need for the precise structures required for its implementation. MS faced daunting technical and organisational challenges, often implementing river basin management in the context of existing water governance structures that varied greatly across the EU, taking significant time and effort to put in place appropriate government agencies (Moss 2012). The Directive’s experimentalist nature might have also contributed to this, particularly considering the failing of the experimentalist approach in some other EU policy areas such as the European Semester (Zeitlin 2016).

But the problems with the Directive had actually started even earlier on. The Directive was the outcome of intense but delicate political negotiations characterised by gradual internal shifts in the governing structures of the EU that left the European Parliament with additional negotiating power and environmental Non-Governmental Organisations with an increasing influence in the discussions (Kaika and Page 2003). With reports of the WFD’s drafting and adoption phases being wrought with political manoeuvrings and profound disagreements, strong opposition from MS during the negotiations seem to have weakened some WFD elements (Kaika 2003). For example, it was the Austrian delegation that insisted and the special status for ‘heavily modified waters’ was introduced; the Spanish, who insisted on a generic terminology in relation to efficient water use and the full-cost recovery for its uses; the German, who disfavoured the case of establishing separate and independent river basin authorities; and the British one that influenced the non-deterioration principle to conform with its existing legislation (Lanz and Scheuer 2001). Exacerbated by shifts in power balance during the negotiation phase, different views and interpretations regarding the WFD’s objectives and exemptions and fears for potential socioeconomic impacts in several EU MS resulted in the final text of the WFD characterised as a hybrid political construct, a weak compromise left open for interpretation (Lanz and Scheuer 2001). Including concepts that are antithetical in their orientation, for example, it sets not only high ecological ambitions but also gives options for exemptions. Similarly, it combines detailed prescriptions and standards with generic frameworks that are related to the German and the Anglo-Saxon philosophies, respectively (Santbergen 2013). From a juridical point of view, the WFD has been said to be one of the most complicated and hard to interpret pieces of EU environmental legislation (Santbergen 2013). Some of its terminological vagueness and the ambiguous wording could be attributed to the subsidiarity principle, a result not only of its troubled negotiation phase but also according to some a strategic move, with objectives and exemptions undefined on purpose in order to be exploited during its implementation (Boeuf et al. 2016).

The CIS process might have established a dialogue with stakeholders and aimed to increase their understanding of the Directive, but many of its recommendations have also been perceived as ambiguous and not very ambitious, often deviating from ‘best practices’ and potentially undermining the spirit of the WFD (WWF and EEB 2004). The consensus-based nature of CIS decision-making, described by ‘a lowest common denominator’ attitude, often turned to be more of a compromise between the need for compliance and the need to adhere to the WFD principles (Korkea-aho 2015).

Conventional practices of centralised decision-making and reductionist thinking dominated implementation efforts (Liefferink et al. 2011; Moss 2004; Nielsen et al. 2013), as the application of the WFD paradigm was met with significant resistance from both the dominant values and interests of previous management approaches (Pahl-Wostl et al. 2011). Authorities carrying out the monitoring were often unwilling to change from their usual practices (Hering et al. 2010), and as a result, the transition from established institutions and governance regimes had been minor with most MS following a rather managerial style, implementing public participation and river basin management structures in the context of established routines of environmental decision-making (Jager et al. 2016). This often turned implementation into a '*tick list*' of compliance against some sets of standards and a range of other discipline-specific management goals—generally without the all-important linkages to address how these different ecosystem parts interact in contrast to the aspirations of the inherently systemic WFD (Everard 2011).

Appreciation of some of the Directive’s innovations and overall philosophical approach remained limited and often shaken by misunderstandings of some of its core principles (Voulvoulis et al. 2017). Countries with well-established water management systems found the process challenging, let alone transitioning ones lacking such a culture (Alexopoulou et al. 2005; Collins et al. 2012). Lack of acceptance and inertia by stakeholders also became obstacles to the implementation (European Commission 2015).

## Discussion

Assessing the effectiveness of the WFD as a policy tool might be more complex and challenging than one might expect. It is not about evaluating the Directive’s success based on the water quality improvements it delivered (or did not) but assessing if the Directive has delivered what it really aimed to achieve. Have the WFD’s inherent interpretation ambiguities triggered or constrained the delivery of a coherent implementation of the IRBM paradigm? And has the perceived autonomy offered by its experimental nature (Wiersema 2008) ended up not being empowering but a restricting gap between law and the practice of governance? (Scott and Holder 2005). And was it because the IRBM paradigm had not been clearly defined that this flexibility did not facilitate the adoption of the systems thinking required to inform the type of participatory ecological design expected by the WFD? And if not, why its approach has been more often criticised for being vague rather seen as flexible (Baaner and Josefsson 2011; Moss 2008).

It is also clear that most implementation efforts applying the river basin approach failed to appreciate the non-linearity of the system, the interdependencies between water and other systems like food and energy production and resource extraction. Such interdependencies have emergent properties that are multidimensional, difficult to quantify and predict (Berkes and Ross 2016; Everard and Powell 2002; Pahl-wostl 2009; Parkes and Horwitz 2009; Surridge and Harris 2007). Research shows that, for a start, the IRBM paradigm means different things to different people and often depending on context. IRBM has been interpreted in multiple ways, particularly considering how it has been aligned to existing patterns of legal pluralism. From integrated water resources management as an idea in international and national fora to its translation and adoption by the WFD into national contexts and the practice of IRBM at the catchment level, the harmonised transposition of its principles during the implementation was meant to serve as the key instrument for MS to understand problems and take appropriate action to reduce pressures and improve ecological status. However, policy discourse, translation problems, institutional bricolage and agency practices were some of the reasons, to name a few, unearthing the convergences and divergences in its various understandings and applications. The WFD might be common sense to water managers, but overall as a policy tool, its ‘integrated’ approach by definition implies a level of systemic thinking quite different to traditional practices. It demands a fundamental shift in water resources planning and management: a shift towards managing the catchment as one system, where water quality improvements are delivered with the system improving its state. Adaptive management requires understanding the ecosystem as a whole before efforts to manage it. This implies a focus on the bioregion especially when such a region crosses multiple administrative borders (Huitema et al. 2009).

Socio-hydrological systems are reflexive, adaptive, non-linear and complex and have feedback loops, emerging properties and non-predictable responses to management interventions (Del Moral et al. 2014), therefore not adopting a systems approach could simply mean managing them all the same. The fragmentation effect of ‘systems’ of the WFD implementation to date has obscured the broader focus on sustainable outcomes. Implementing the WFD in a way that allows a transition to the IRBM paradigm requires real transformational change. It requires integration of disciplines, analyses and expertise, combining hydrology, hydraulics, ecology, chemistry, soil sciences, technology, engineering and economics to assess current pressures and impacts on water resources and identify measures for achieving the environmental objectives of the Directive in the most cost-effective manner (European Communities 2003b). The WFD approach, accounting for resource efficiency, resilient ecosystems and human wellbeing, requires interdisciplinary research for the IRBM paradigm shift necessary. With the concept almost hijacked by ecologists and with a reductionist conception of nature prevailing during the implementation, what is required is true collaboration for understanding and managing the water environment as a complex system (Zalewski 2015). Supported by collaborative knowledge production processes crossing the multiple boundaries between the various groups involved in river basin management, this requires engagement of scientists from different scientific backgrounds, stakeholders with different interests, policymakers from different policy sectors and politicians from different political parties (Slob and Duijn 2014).

The upcoming WFD review in 2019 offers the opportunity to break this paradigm by focussing on the functionality of freshwater resources and their relation to the catchment and its socio-economic aspects. It represents a unique opportunity to allow the Directive to deliver its systemic intent. There is a need for all actors involved in the implementation of the WFD from policy-makers and catchment managers to the scientists and civil community to return to the initial aspirations of the WFD, revisit the concepts it embraced and explore ways to operationalise them in order for the WFD to reach its full potential. Unless it is recognised what the WFD aimed to deliver and what approach it adopted for this, there is a potential risk that its flexible and experimental nature could be addressed as the main source of concern, leading to an even more compliance-driven approach.

Implementing the WFD without understanding how it works, why it was introduced and reviewing it out of this context, there is a clear risk that even its core principles will be subject to several multi-interpretations and definitions, with its holistic and integrated approach observed as ambiguity. Implementing the WFD without the paradigm shift towards IRBM will not trigger the rule changes the Directive was introduced to initiate, and the actor networks of the water policy domain will remain overshadowed by governmental authorities and experts, isolated from networks of other policy domains. Implementing the WFD simply to avoid fines or to keep things as they are using intrinsic exemption options and conditions would not enable the WFD to reach its full potential. The 2019 WFD review might be the last chance to recognise and allow the paradigm shift that the Directive was introduced for, with the review being the Directive’s last attempt to safeguard its mission.

## Electronic supplementary material

Supplementary Information
