# Supplementary material for: The Transition of EU Water Policy Towards the Water Framework Directive’s Integrated River Basin Management Paradigm
Source: Environ Manage. 2018 Jul 9;62(5):819–31. doi: 10.1007/s00267-018-1080-z (PMC6208820; doi:10.1007/s00267-018-1080-z)
Supplement: Supplementary file 1 — Supplementary Information [file 267_2018_1080_MOESM1_ESM.docx]

# Supplementary Materials

# The transition of EU water policy towards the Water Framework Directive’s Integrated River Basin Management Paradigm

Theodoros Giakoumis ^a^ and Nikolaos Voulvoulis ^a^*

^a^Centre for Environmental Policy, Imperial College London, London SW7 2AZ, United Kingdom

*Corresponding author. Tel.: +44 020 7594 7459; Fax: +44 020 7594 9334.

Email: [n.voulvoulis@imperial.ac.uk](mailto:n.voulvoulis@imperial.ac.uk)

Supplemental data includes three tables (S1, S2, S3)

**Table S1** Major Policy Actions and developments that led to the adaptation of the WFD

| **Year** | **Activity** |
| --- | --- |
| 1972 | The EU environmental policy was formally founded through the European Council declaration made in Paris. EU water legislation began with the setting of standards for rivers and lakes used for drinking water abstraction. |
| 1973 | The EU adopts its First EAP, based on the ideas that prevention is better than cure and the ‘polluter pays’ principle. The first environment ministries were established. |
| 1976 | Bathing waters; Dir. 76/464/EEC on pollution caused by discharges of certain dangerous chemicals into the aquatic environment. |
| 1977 | The Second EAP was adopted. It advocated quality values for water. |
| 1978 | Fish waters (Council Directive 78/659/EEC) on the quality of fresh waters aimed to protect or improve waters in order to support fish life. |
| 1979 | Council Directive 79/923/EEC on the protection of shellfish waters enters into force. It was amended by the Council Directive 91/692/EEC (further amended by Council Regulation 1882/2003/EC). |
| 1980 | Council Directive 80/68/EEC for discharges to the ground water enters into force |
|  | Council Directive 80/778/EEC enters into force but was repealed with effect from five years after the entry into force of Directive 98/83/EC. |
| 1982 | The Third EAP was adopted. It promoted a shift towards an emission-oriented approach. |
| 1986 | Single European Act (SEA) was the first major revision of the 1957 Treaty of Rome. |
| 1987 | The Fourth EAP entered in to force and initiated a “sectoral approach”, analysing the impact of strategic economic sectors on the environment. |
| 1991 | Urban Wastewater Treatment Directive (91/271/EEC), aimed to protect the environment from the adverse effects of untreated urban waste water discharges and discharges from certain industrial sectors; Sets standards for collection and treatment of wastewater from different sizes of agglomerations; Designate areas sensitive to nutrient pollution |
|  | Nitrates Directive (91/676/ECC), aimed to protect water quality across Europe by preventing nitrates from agricultural sources polluting ground and surface waters and by promoting the use of good farming practices. |
| 1992 | The Habitats Directive (92/43/EEC) was adopted and aimed to achieve favourable conservation status for selected habitat types and species by designating special protection areas. |
|  | Maastricht Treaty legitimises the subsidiarity principle. |
| 1993 | The Fifth EAP entitled “Towards Sustainability” was adopted. |
|  | The proposal for a Directive on the Ecological Quality of Water (COM 93 680), a first attempt for a framework policy on water with many similarities to the WFD. It proposed a more comprehensive approach, but relied heavily on the principle of subsidiarity, allowing for variable implementation, and was criticised for not introducing strong enough measures.  "Ecological water quality" was defined as "an overall expression of the structure and function of the biological community taking into account natural physiographic, geographical and climatic factors as well as physical and chemical conditions, including those resulting from human activities. The aesthetics of the area should also be taken into account". |
| 1994 | The Commission proposed a Directive (COM(93)680) on the ecological quality of surface waters as a more comprehensive approach, but relied heavily on the principle of subsidiarity, allowing for variable implementation, and was criticised for not introducing strong enough measures. |
| 1995 | In June 1995, the Council and the Environment Committee of the European Parliament called for a fundamental review of Community water policy. In November of that same year, the European Environment Agency produced its report “Environment in the European Union – 1995” in which it found a need for action to protect Community waters in both qualitative and quantitative terms. The next month, December of 1995, the Council adopted a resolution that required the Commission to draft a proposal for a new Directive on sustainable water policy. |
| 1997 | The Commission presents its Communication on European Community Water Policy (COM(96)59) proposing the WFD. This communication evaluated previous water policies, defined objectives for a new policy framework, and gave recommendations for future action. |
| 1997 | The Commission produced a first proposal for a WFD (COM(97)49). |
| 1998 | The UK Presidency placed a WFD proposal on the Council agenda and produced a significantly modified version. |
| 1999 | The Amsterdam treaty came into force, amending the Treaty of the European Union, enabling the WFD proposal being adopted by co-decision, as Parliament gains equal legislative power with the Council of Ministers. |
| 2000 | Directive 2000/60/EC known as the WFD of 23 October 2000 establishing a framework for Community action in the field of water policy) was signed. |

**Table S2** Milestones in WFD implementation and policy progress and developments since 2000

| **Year** | **Milestones in WFD implementation, progress and policy developments** |
| --- | --- |
| 2000 | WFD entered into force, (WFD Art. 25). |
| 2001 | The Council adopted Decision establishing a list of priority substances in the field of water policy, formally proposed at the beginning of 2001 (COM(2001)17) and formed Annex X of the WFD. The priority list of 33 substances is derived from an initial survey of other ‘lists’, including those in Directive 76/464/EEC, the OSPAR and HELCOM Conventions, etc (WFD Art 16). |
|  | A Common Implementation Strategy is developed as a response to the complex technical issues in implementing the Directive. |
| 2002 | The Sixth EAP was elaborated through a co-decision procedure of the European Parliament and of the Council. Its main objective in relation to water management was to ensure the full implementation of the WFD through the characterisation of river basin districts, the environmental pressures and impacts and economics of water uses. It promoted the full integration of environmental protection requirements into all Community policies and actions and provided the environmental component of the Community’s strategy for sustainable development. |
| 2003 | The deadline for transposition in national legislation, (WFD Art. 23), was poorly met and in many cases inadequate. The conformity assessment in 2007 of transposition of Articles 4, 4.7, 9 and 14 only 3 MS have been successful 2 partially having missing elements or issues that require further analysis (European Commission, 2007). Infringement cases for Greece, Italy and Luxemburg were still open by 2007. |
|  | The deadline for Identification of River Basin Districts and Authorities, (WFD Art. 3) was largely met by most EU 25 MS. The Commission launched infringement cases of "non-communication" against Belgium, Denmark, France, Greece, Italy, Malta, Poland, Spain and Sweden since they failed to submit any report in time. All cases, with the exception of Spain, have been resolved, most of them already in 2004, and closed in the meantime. By 2007, the implementation of Article 3 was largely complete across EU27. This has resulted in the establishment of 110 river basin districts (RBDs) across the EU. |
|  | The Commission published the first eight CIS guidance documents on technical aspects, (WFD Art. 20). |
| 2004 | Characterisation of river basin: pressures, impacts and economic analysis, (WFD Art. 5). Only 8 MS of EU25 have submitted their Article 5 report in time and 9 additional MS have sent their report within three months after the deadline of 22 March 2005. The Commission started legal action on "non-communication" against Spain, Portugal, Greece and Italy. The economic analysis was the weakest part of the Article 5 reports. This concerned in particular the definition of water services, and the information for the calculation of recovery of costs of water services, particularly information on environmental and resource costs and information on sectors to be affected by cost recovery (European Commission, 2007b). |
| 2006 | Deadline for establishing of monitoring networks, (WFD Art. 8); All MS have reported with the exception of Greece and Malta (for surface water monitoring programmes). |
| 2007 | The Communication ‘Addressing the challenge of water scarcity and droughts’ was published. Seven policy options were identified for tackling water scarcity and drought issues:  A) Putting the right price tag on water, B) Allocating water and water-related funding more efficiently, C) Improving drought risk management, D) Considering additional water supply infrastructures, E) Fostering water efficient technologies and practices, F) Fostering the emergence of a water-saving culture in Europe and G) Improve knowledge and data collection |
|  | The Commission published its first report (COM(2007)128) on the implementation of the WFD, summarizing the results of reports made by the MS under Article 5. It was accompanied by a more detailed Staff Working Document (SEC(2007)362), based on environmental and economic analysis of water bodies that according to Article 5 were required to be produced by 2004. The striking features of the report were the number of water bodies ‘at risk’ and data gaps. |
|  | The Floods Directive (FD) was adopted in 2007 with the aim of managing and reducing the risks that significant floods pose to human health, the environment, cultural heritage and economic activity. It prescribes an active involvement of all interested stakeholders in the process, requiring MS to map the extent of floods, to assess the people and assets at risk in these areas and to take adequate and coordinated measures to reduce this flood risk, as detailed in Flood Risk Management Plans. These plans should take into account the relevant environmental objectives of the WFD. |
| 2008 | The Directive 2008/105/EC on quality standards in water was adopted. Annex II of 2008/105/EC replaced Annex X of the WFD. It establishes environmental quality standards for surface waters (or exceptionally sediments and/or biota) for 33 ‘priority substances’ and 8 other pollutants in order to achieve the objectives of the WFD. |
|  | MS should present draft river basin management plan, (WFD Art. 13) |
| 2009 | The White Paper “Adapting to climate change: Towards a European framework for action”, set out a framework to reduce the EU’s vulnerability to the impact of climate change. It fosters the development of strategies for the management and conservation of water. |
|  | The Commission published the second implementation report on monitoring networks (COM(2009)156), for all MS except from Greece which did not report and Malta, which did not report on surface water monitoring programmes. Overall, there was a good monitoring effort across the EU, with more than 107,000 monitoring stations reported for monitoring of surface water and groundwater. However, gaps were detected in individual river basin districts or individual water categories. For instance, there were still many river basin districts where the necessary assessment methods for biological quality elements were not yet in place. This was particularly true in the countries that joined the EU in 2004 and 2007. |
|  | Deadline to finalise river basin management plan including programme of measures, (WFD Art. 13 & 11). Although drafts were made available for public consultation in 17 MS (many of those not available were in southern MS) in 2009. The most frequently used exemptions were extension of deadlines and use of less stringent objectives. |
| 2010 | The deadline for introducing pricing policies (WFD Art. 9), was not met by MS, with proposals on water pricing not often identified in the RBMPs. |
| 2011 | The EU Resource Efficiency Roadmap (COM(2011) 571 final) sets a long term framework to guarantee that several areas such as energy, climate change, research and innovation, industry and environmental policy will lead to a resource efficient Europe. |
|  | The EU Biodiversity Strategy 2020 (COM(2011) 244 final) sets out 6 targets and 20 actions aiming to halt the loss of biodiversity and ecosystem services in the EU by 2020. |
|  | The Commission stated that 23 MS and Norway had adopted their River Basin Management Plans, but for 4 MS (Belgium, Greece, Portugal and Spain) the consultations were on-going or had not even started. |
| 2012 | Deadline to make operational programmes of measures (WFD Art. 11). |
|  | The third implementation report on the River Basin Management Plans was adopted (COM(2012)670). It shows that more efforts are needed to ensure the achievement of the WFD objectives in the 2015, 2021 and 2027 planning cycles. The assessment of the RBMPs indicates that progress towards the objective of good status by 2015 is expected, but it will not be achieved for a significant proportion of water bodies. |
|  | The Commission published the “Blueprint for Europe's water resources”, integrating the results of a policy review concerning: water scarcity and droughts; an analysis of the implementation of river basin management under the WFD; a review of the vulnerability of environmental resources to climate change impacts and man-made pressures; and a review of the whole of the EU’s water policy framework in the light of the European Commission’s ‘better regulation’ approach. |
| 2013 | The Directive 2013/39/EU dealing with Priority Substances under the WFD was amended and updated in the original WFD and the Environmental Quality Standards Directive (2008/105/EC). |
| 2013 | The Seventh EAP, entitled ‘Living well, within the limits of our planet’ (DECISION No 1386/2013/EU) was adopted. It will be guiding European environment policy until 2020 and builds on policy initiatives in the Europe 2020 Strategy and the EU Strategy for Sustainable Development. It reiterates the need of full implementation of the WFD and reconfirms objectives and measures proposed in the *Blueprint*. It gives a long-term direction, as it sets out a vision of where the EU wants to be by 2050. The biggest consuming sectors, such as energy and agriculture, should be encouraged to prioritise the most resource-efficient use of water. Progress will be facilitated by accelerated demonstration and rolling out of innovative technologies, systems and business models building on the Strategic Implementation Plan of the European Innovation Partnership on Water. |
| 2015 | The fourth WFD implementation report on the programmes of measures and the Flood Directive was adopted (COM/2015/0120). Surface water bodies expected to be in ‘good status’ have increased from 43% in 2009 to 53% in 2015. |
|  | The first management cycle ended. |
|  | The first official deadline to meet environmental objectives (WFD Art. 4) has not been achieved. Progress made has not gone far enough or been delivered fast enough and despite the economic climate, efforts must accelerate to achieve the original ambitions since water is essential for people, nature and the economy. |
|  | The second river basin management plan & first flood risk management plan begins. |
| 2021 | The second management cycle to end (WFD Art. 4 & 13). |
| 2027 | Third management cycle to end. The final deadline for meeting objectives (WFD Art. 4 & 13). |

Table S3 Summary table of WFD related infringement cases examples

| **Judgment of the Court** | **State** | **Failure of a Member State to fulfil obligations of Directive 2000/60/EC - Framework for Community action in the field of water policy** |
| --- | --- | --- |
| (Sixth Chamber) of 12 January 2006. | EC v Portuguese Republic | Failure to transpose within the prescribed period. |
| (Sixth Chamber) of 12 January 2006. | EC v Italian Republic | Failure to transpose within the prescribed period. |
| (Third Chamber) of 30 November 2006. | EC v Grand Duchy of Luxemburg | Failure to notify implementing measures - Obligation to adopt framework legislation in national law - None - Incomplete implementation of or failure to implement Articles 2, 7(2) and 14. |
| (Eighth Chamber) of 18 December 2007. | EC v Italian Republic | Articles 5(1) and 15(2) - River basin district - Summary report and analyses - Communication thereof - None. |
| (Seventh Chamber) of 31 January 2008. | EC v Hellenic Republic | Protection and management of water - Failure to undertake the analyses required - Failure to submit the summary reports required. |
| (Seventh Chamber) of 7 May 2009. | EC v Kingdom of Spain | Designation of competent authorities for hydrographic districts. |
| (First Chamber) of 22 December 2010. | EC v Republic of Malta | Articles 8 and 15 - Status of inland surface water - Establishment and making operational of monitoring programmes - Failure - Submission of summary reports on those monitoring programmes - Failure. |
| (Fifth Chamber) of 21 June 2012 | EC v Portuguese Republic | RBMPs — Publication and notification to the Commission — None — Information and consultation of the public on the envisaged management plans — None |
| (Fifth Chamber) of 24 May 2012. | EC v Kingdom of Belgium | RBMPs- Publication and notification to the Commission - Lack - Public information and consultation concerning the draft management plans - Lack. |
| (Sixth Chamber) of 19 April 2012. | EC v Hellenic Republic | RBMPs - Publication - Public information and consultation - Failure to notify the Commission. |
| (Sixth Chamber) of 4 October 2012. | EC v Kingdom of Spain | RBMPs - Publication and reporting to the Commission - Public information and consultation - Failure to undertake. |
| (Fifth Chamber), 24 October 2013 | EC v Kingdom of Spain | Transposition of Articles 4(8), 7(2), 10(1) and (2) of and sections 1.3 and 1.4 of Annex V to Directive 2000/60 — Intra-communal and inter-communal river basins — Article 149(3) of the Spanish Constitution — Supplementing clause |
| (Second Chamber) of 11 September 2014. | EC v Federal Republic of Germany | Recovery of the costs for water services - Concept of ‘water services’. |
| (Eighth Chamber) of 6 November 2014 | EC v Kingdom of Denmark | RBMPs— Publication — Failure to notify the European Commission |
| (First Chamber) of 4 May 2016 | EC v Republic of Austria | Article 4(1) — Prevention of deterioration of the status of bodies of surface water — Article 4(7) — Derogation from the prohibition of deterioration — Overriding public interest — Authorisation to construct a hydropower plant on the Schwarze Sulm River (Austria) — Deterioration of the water status |
| (Sixth Chamber) of 30 June 2016 | EC v Republic of Poland | Monitoring of the ecological status and the chemical status of surface waters — RBMPs |
